# Integrative analyses of genes and microRNA expressions in human trisomy 21 placentas

**DOI:** 10.1186/s12920-018-0361-y

**Published:** 2018-05-09

**Authors:** Ji Hyae Lim, You Jung Han, Hyun Jin Kim, Moon Young Kim, So Yeon Park, Youl-Hee Cho, Hyun Mee Ryu

**Affiliations:** 1 Laboratory of Medical Genetics, Medical Research Institute, Cheil General Hospital and Women’s Healthcare Center, Seoul, South Korea; 2 Department of Medical Genetics, College of Medicine, Hanyang University, 222, Wangsimni-ro, Seongdong-gu, Seoul, 04763 South Korea; 3 Department of Obstetrics and Gynecology, Cheil General Hospital and Women’s Healthcare Center, Dankook University College of Medicine, 1-19, Mookjung-dong, Chung-gu, Seoul, 100-380 South Korea

**Keywords:** Gene expression, MicroRNA, Placenta, Trisomy 21, Whole genome

## Abstract

**Background:**

The most frequent chromosomal aneuploidy is trisomy 21 (T21) that is caused by an extra copy of chromosome 21. The imbalance of whole genome including genes and microRNAs contributes to the various phenotypes of T21. However, the integrative association between genes and microRNAs in the T21 placenta has yet to be determined.

**Methods:**

We analyzed the expressions of genes and microRNAs in the whole genomes of chorionic villi cells from normal and T21 human fetal placentas based on our prior studies. The functional significances and interactions of the genes and microRNAs were predicted using bioinformatics tools.

**Results:**

Among 110 genes and 34 microRNAs showing significantly differential expression between the T21 and normal placentas, the expression levels of 17 genes were negatively correlated with those of eight microRNAs in the T21 group. Of these 17 genes, 10 with decreased expression were targeted by five up-regulated microRNAs, whereas seven genes with increased expression were targeted by three down-regulated microRNAs. These genes were significantly associated with hydrogen peroxide-mediated programmed cell death, cell chemotaxis, and protein self-association. They were also associated with T21 and its accompanying abnormalities. The constructed interactive signaling network showed that seven genes (three increased and four decreased expressions) were essential components of a dynamic signaling complex (*P* = 7.77e-16).

**Conclusions:**

In this study, we have described the interplay of genes and microRNAs in the T21 placentas and their modulation in biological pathways related to T21 pathogenesis. These results may therefore contribute to further research about the interaction of genes and microRNAs in disease pathogenesis.

## Background

Trisomy 21 (T21) is the most frequent chromosome aneuploidy affecting 1 in 700 live births [1]. Individuals with T21 have an increased risk of various congenital abnormalities, including eye, cardiac, gastrointestinal, renal and urinary tract defects [2]. These defects are generally considered to originate from gene dosage imbalance between the trisomic genes on chromosome 21 and the disomic genes on other chromosomes. Therefore, studies of T21 have focused mainly on expression levels of the chromosome 21-derived genome in various tissues from subjects with the condition [3–5]. However, the severity and incidence of those phenotypic abnormalities are variable within the T21 population, possibly due to the genetic and epigenetic backgrounds of each individual.

MicroRNAs (miRNAs) are small (18~ 25-nucleotide-long) non-coding endogenous RNAs. They regulate expression of genes at the post-transcriptional level by regulating mRNA stability and translation [6–9]. More than 1000 miRNAs are expected to participate in regulating over 60% of all the genes [10, 11]. Hence, miRNAs seem to be involved in almost all cellular processes, such as cell apoptosis, differentiation, development, and proliferation [12, 13]. Moreover, changes in their expression levels are reported in various human diseases such as cancer, cardiovascular disease, mental retardation, fetal growth restriction, Alzheimer’s disease, and T21 [14–19]. For this reason, great attentions are currently devoted to miRNA research.

In our previous studies, we profiled expression levels of genome-wide miRNAs and genes in placental samples from normal and T21 fetuses using microarray analyses [20, 21]. Our results demonstrated that 34 miRNAs (16 up-regulated and 18 down-regulated) and 110 genes (77 up-regulated and 33 down-regulated) were significantly differentially expressed in the T21 placenta compared with that in normal placentas. Moreover, these miRNAs targeted 76 genes on chromosome 21, suggesting a relationship between genetic and epigenetic changes in the placentas of fetuses with T21. However, the association between genes and miRNA expressions in the whole genome has not yet been determined in the T21 placenta, and the functional significances of these genetic and epigenetic interactions are also unclear. Therefore, an integrative investigation of human genes and miRNAs in the whole genome might be important in understanding the complex genetic-epigenetic mechanisms involved in the pathogenesis of T21 associated abnormalities.

The miRNAs and genes differentially expressed between the placentas of normal and T21 fetuses were found in our previous data [20, 21], and in this study we could identify genes showing a negative correlation with miRNAs, and explored the biological function and molecular pathways of the identified genes using various bioinformatics tools.

## Methods

### Study subjects

The placenta cells were collected by the chorionic villi sampling (CVS) from first-trimester pregnant women. The written informed consents were obtained from participants in compliance with the Declaration of Helsinki. The institutional review board approval was received from the Ethics Committee at Cheil General Hospital (#CGH-IRB-2011-85). The fetal karyotype was analyzed by standard protocols using the Giemsa banding procedure. All trisomy samples used in this study were completely T21, and all normal samples were completely euploid.

#### Expression profiling of genome-wide whole genes and miRNAs

Expression of genes and miRNAs in the whole genomes was analyzed based on our previous studies [20, 21]. In brief, total RNA was extracted from normal and T21 fetal placentas. An RNA quantity, quality, and integrity number were measured by an Agilent 2100 Bioanalyzer (Agilent Technologies, CA, USA) and a NanoVue Plus spectrophotometer (GE Healthcare, London, UK). Expression profiles of whole genes were determined using the Affymetrix GeneChip Human Genome U133 Plus 2.0 Array (Affymetrix Inc., Santa Clara, CA, USA) [21]. The miRNAs expressions were profiled using Human miRNA Microarray kit, 8 × 60 K (based on miRBase release 16.0, Agilent Technologies) [20]. Differences in expressions of genes and miRNAs between the T21 and normal groups were considered significant at a *P-*value of < 0.05. The Benjamini–Hochberg procedure was used to set the false discovery rate (FDR) at 0.05 [22].

#### Functional annotation of the candidate genes

All target genes of the 34 miRNAs differentially expressed in T21 were compared with 110 genes differentially expressed in T21 using the VENNY tool (http://bioinfogp.cnb.csic.es/tools/venny_old/index.html). The genes showing a negative correlation with miRNAs in terms of expression were selected as candidates for functional annotation. The web-based gene set analysis toolkit (http://www.webgestalt.org/webgestalt_2013) was used for gene ontology (GO) analysis, Kyoto encyclopedia of genes and genomes (KEGG) pathway analysis, and disease-associated analysis. The Search Tool for the Retrieval of Interacting Genes database (STRING; http://version10.string-db.org/) is a database to retrieve and display protein-protein interactions, including both physical and functional interactions. STRING was used to analyze an interaction of candidate genes.

#### Statistical analyses

The clinical characteristics were analyzed by the Mann-Whitney U-test and χ^2^-test. In all tests, a value of *P* < 0.05 was considered statistically significant (SPSS Inc., Chicago, IL, USA).

## Results

Study subjects were constructed with 10 women with euploid fetuses and seven women with T21 fetuses. The CVS from five euploid and three T21 placentas were used for expression profiling of genome-wide whole genes [20], and those from the other five euploid and four T21 placentas were used for miRNA expression analysis [21]. Table 1 shows the clinical characteristics of the subjects. At the time of CVS, there were no significant differences between the two groups with regard to maternal and fetal characteristics (*P* > 0.05 for all).

**Table 1:** Clinical characteristics of the study population

Characteristics	mRNA profiling			microRNA profiling			Total subjects		
	Trisomy 21 (*n* = 3)	Normal (*n* = 5)	*P* value	Trisomy 21 (*n* = 4)	Normal (n = 5)	*P* value	Trisomy 21 (*n* = 7)	Normal (*n* = 10)	*P* value
Maternal Age (years)	34.6 ± 3.8	37.4 ± 3.9	0.853	30.3 ± 2.2	34.2 ± 3.1	0.628	32.1 ± 3.6	35.8 ± 3.7	0.062
Gestational age (weeks)	12.0 ± 0.0	12.2 ± 0.4	0.092	12.2 ± 0.4	12.4 ± 0.7	0.071	12.1 ± 0.4	12.3 ± 0.6	0.454
Body mass index (kg/m^2^)	22.3 ± 3.7	24.4 ± 6.2	0.545	21.8 ± 3.3	21.5 ± 1.2.2	0.874	22.0 ± 3.2	22.9 ± 4.7	0.659
Gravidity	2.7 ± 1.2	2.8 ± 0.8	0.446	1.8 ± 1.0	3.0 ± 1.6	0.210	2.1 ± 1.1	2.9 ± 1.2	0.201
Nuchal translucency (mm)	3.7 ± 1.4	4.9 ± 1.9	0.402	4.2 ± 0.8	3.3 ± 1.5	0.377	4.0 ± 1.0	4.1 ± 1.8	0.862
Nullipara	2	1	0.146	2	1	0.524	4	2	0.162
Gender-ratio (female:male)	1:2	1:4	1.000	1:3	1:4	1.000	2:5	2:8	1.000

**Fig. 1:**
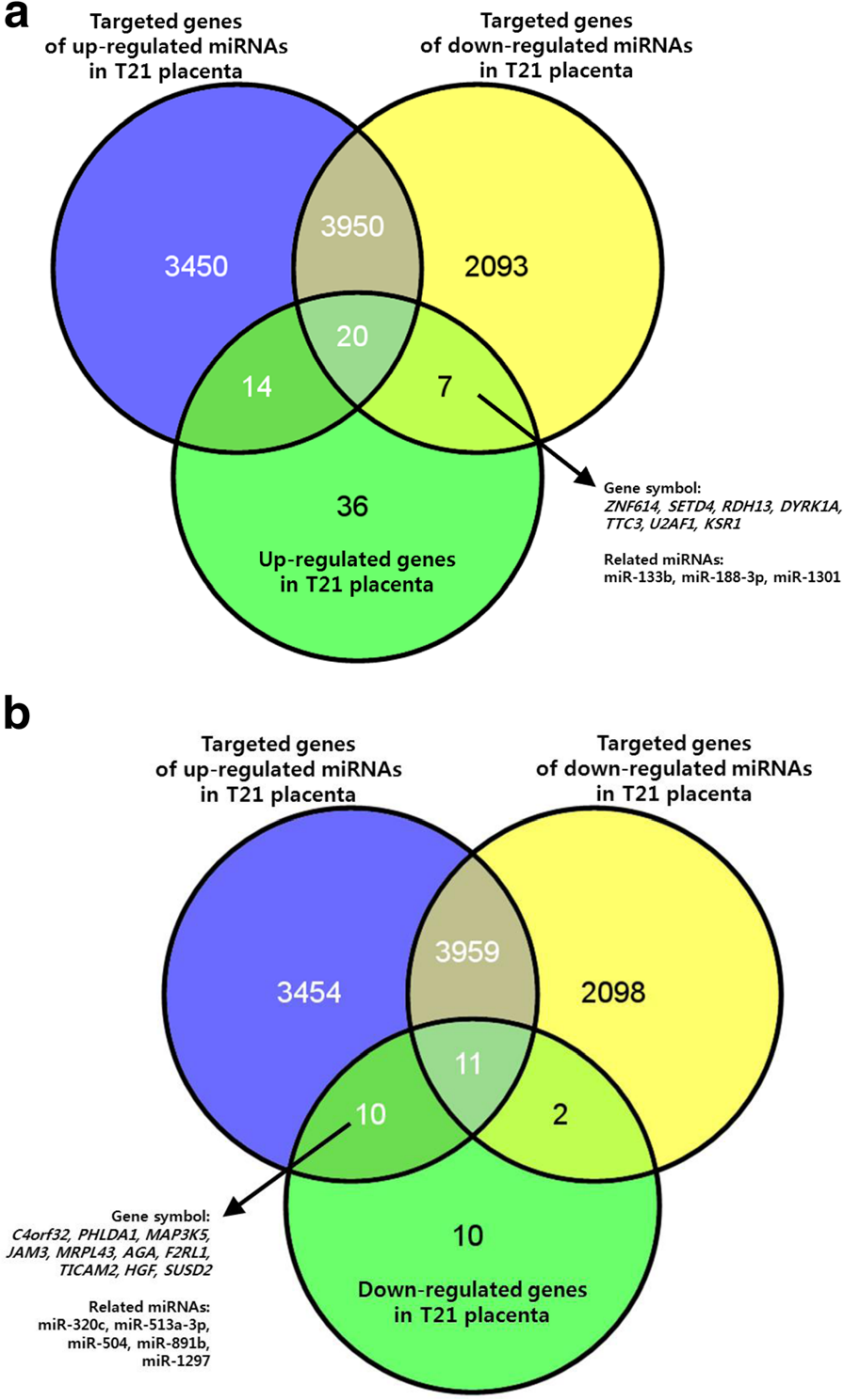
Negative correlation between gene and miRNA expression levels. **a**) Venn diagram showing the overlap of up-regulated genes and down-regulated miRNAs with significant changes in the T21 placenta. **b**) Venn diagram showing the overlap of down-regulated genes and up-regulated miRNAs with significant changes in the T21 placenta

Based on our previous studies, we had analyzed the expression levels of over 47,000 genes and 1349 miRNAs in the whole human genome. Thirty-four miRNAs were differentially expressed (16 up-regulated and 18 down-regulated) in the T21 placentas relative to that in the normal placentas. The number of predicted target genes of the up-regulated and down-regulated miRNAs was 7421 and 6058, respectively (Fig. 1). The 110 genes showing significant differential expression between the T21 and normal placentas were identified, of which 77 genes were up-regulated and 33 genes were down-regulated in the T21 group. The 110 genes and 34 miRNAs were selected for the analysis of genetic-epigenetic association in the T21 placenta. In the analysis of the miRNA-gene associations, 17 genes showed a negative correlation with eight miRNAs in terms of expression (Fig. 1 and Table 2). These 17 genes were selected as candidate genes for the functional annotation.

**Table 2:** Expression changes of microRNA and genes showing negative correlation in the T21 placenta

Expression pattern of mRNAs in the T21 placenta	Gene Symbol	Fold intensity	*P* value	FDR	Chr	microRNAs showing negative correlation				
						mir	Fold intensity	*P* value	FDR	Chr
Up-regulation	*DYRK1A*	1.530	0.013	0.008	21	mir-188-3p	4.301	0.004	0.007	X
	*KSR1*	1.805	0.013	0.006	17	mir-1301	6.571	0.016	0.046	2
	*RDH13*	1.634	0.016	0.018	19	mir-188-3p	4.301	0.004	0.007	X
	*SETD4*	1.696	0.007	0.009	21	mir-1301	6.571	0.016	0.046	2
	*TTC3*	1.678	0.037	0.045	21	mir-133b	5.470	0.001	0.001	6
	*U2AF1*	1.856	0.007	0.025	21	mir-133b	5.470	0.001	0.001	6
	*ZNF614*	1.615	0.043	0.040	19	mir-188-3p	4.301	0.004	0.007	X
Down-regulation	*AGA*	1.520	0.005	0.002	4	mir-513a-3p	4.192	0.013	0.040	X
	*C4orf32*	1.798	0.017	0.015	4	mir-504,	3.848	0.006	0.012	X
						mir-513a-3p	4.192	0.013	0.040	X
						mir-891b	4.407	0.014	0.041	X
	*F2RL1*	1.846	0.006	0.004	5	mir-513a-3p	4.192	0.013	0.040	X
						mir-1297	8.045	0.012	0.036	13
	*HGF*	2.404	0.020	0.014	7	mir-1297	8.045	0.012	0.036	13
	*JAM3*	1.885	0.032	0.013	11	mir-320c	2.558	0.002	0.002	18
	*MAP3K5*	1.617	0.033	0.029	6	mir-513a-3p	4.192	0.013	0.040	X
						mir-891b	4.407	0.014	0.041	X
	*MRPL43*	2.611	0.020	0.016	10	mir-504	3.848	0.006	0.012	X
	*PHLDA1*	1.905	0.027	0.010	12	mir-513a-3p	4.192	0.013	0.040	X
						mir-891b	4.407	0.014	0.041	X
						mir-1297	8.045	0.012	0.036	13
	*SUSD2*	2.324	0.024	0.008	22	mir-1297	8.045	0.012	0.036	13
	*TICAM2*	1.604	0.043	0.033	5	mir-891b	4.407	0.014	0.041	X

rawP: p value from hypergeometric test, adjP: p value adjusted by the multiple test adjustment

In the *in-silico* analysis using the 17 candidate genes, GO annotation and disease association analyses were performed by a statistical hypergeometric test (Table 3). In the “biological process” category of GO annotation, the candidate genes, *HGF* and *MAP3K5*, were significantly associated with hydrogen peroxide-mediated programmed cell death (adj*P =* 0.0008). The *F2RL1, HGF,* and *JAM3* genes were associated with cell chemotaxis (adj*P =* 0.0435). Protein self-association (adj*P =* 0.0172) in the molecular function category of GO annotation was significantly associated with genes, *AGA* and *DYRK1A*. However, none of the candidate genes were associated with the “cellular component” category of GO annotation. The disease associations of the candidate genes are shown in Table 4. The most statistically significant association with candidate genes, *AGA, DYRK1A, SETD4,* and *TTC3*, was found in mental retardation (adj*P =* 0.0014). Besides this, the candidate genes were also significantly associated with T21, neurobehavioral manifestations, chromosome disorders, osteoarthritis, and fibrosis (adj*P <* 0.05 for all).

**Table 3:** GO analysis of identified genes

	Pathway	GeneSymbol	rawP	adjP
BP	Hydrogen peroxide-mediated programmed cell death	*HGF, MAP3K5*	0.000002	0.0008
	Cell chemotaxis	*F2RL1, HGF, JAM3*	0.0003	0.0435
MF	Protein self-association	*AGA, DYRK1A*	0.0004	0.0172

*BP* Biological process, *MF* Molecular function, *GO* gene ontology, *rawP* p value from hypergeometric test, *adjP p* value adjusted by the multiple test adjustment

**Table 4:** Disease association of identified genes

Disease	GeneSymbol	rawP	adjP
Mental Retardation	*AGA, DYRK1A, SETD4, TTC3*	0.0001	0.0014
Down Syndrome	*DYRK1A, SETD4, TTC3*	0.0004	0.0028
Trisomy	*DYRK1A, TTC3*	0.0016	0.0056
Neurobehavioral Manifestations	*DYRK1A, SETD4, TTC3*	0.0016	0.0056
Chromosome Disorders	*DYRK1A, SETD4, TTC3*	0.0021	0.0059
Osteoarthritis	*F2RL1, JAM3*	0.0036	0.0084
Fibrosis	*F2RL1, HGF*	0.0098	0.0196

rawP: *p* value from hypergeometric test, adjP: *p* value adjusted by the multiple test adjustment

**Fig. 2:**
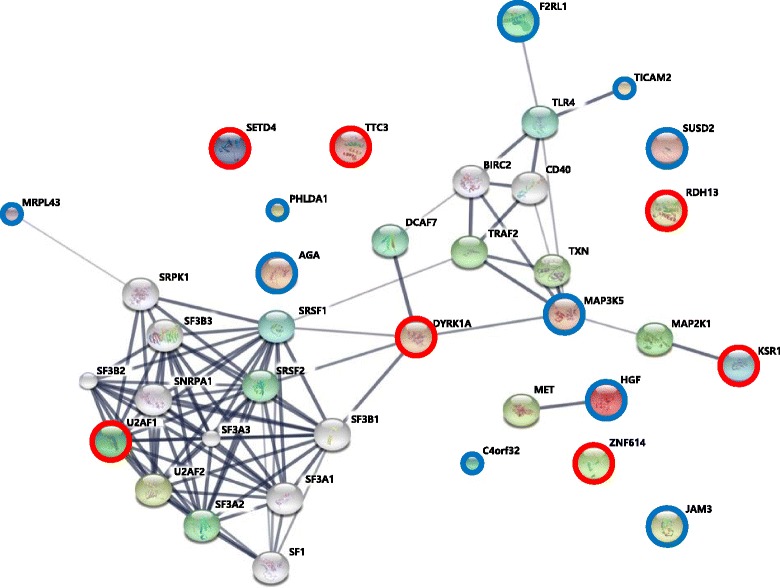
Interaction networks of candidate genes that are differentially expressed in T21 placentas by targeting of miRNAs. The interaction network of 17 genes by targeting of eight miRNAs that are differentially expressed in the T21 placenta (*P* = 7.77e-16). Red circles and blue circles show up-regulated genes and down-regulated genes, respectively

An interaction of the candidate genes was predicted by STRING tool (Fig. 2). The list of the identified candidate genes was used to reveal their functional interactions. Each node represents a protein, and each edge represents an interaction. Thicker lines represent stronger associations. On the basis of 17 genes showing a negative correlation with miRNAs, the part of the dynamic signaling complex in the constructed interaction network included three up-regulated (*U2AF1*, *DYRK1A*, and *KSR1*) and four down-regulated genes (*MRPL43*, *F2RL1*, *TICAM2*, and *MAP3K5*) (Fig. 2C).

## Discussion

To date, most studies have confirmed a gene dose effect of chromosome 21 in T21, because the main etiology of T21 has been known as an imbalance dosage of genes on chromosome 21 [23, 24]. However, the downstream consequences of T21 are complex. In other words, gene expression imbalances of chromosome 21 affect transcription factors, chromatin remodeling proteins, or related molecules on other chromosomes [25, 26]. These suggest that genomic dosage changes of chromosome 21 in T21 could be relatively subtle or massively disruptive to various genes on other chromosomes. Therefore, as well as primary gene dosage effects in pathogenesis T21, secondary (downstream) effects of disomic genes on other chromosomes are also likely to have a major role in T21. In gene expression process of the human genome, the miRNAs affect as key regulators. They induce mRNA degradation of target genes by perfect binding to target mRNAs or inhibit their translation by imperfect complementary binding to the 3′ untranslated region [27, 28]. Moreover, these miRNAs are involved in the occurrence and development of various diseases [14–19]. Therefore, understanding the whole genomic changes that contribute to the various phenotypes of T21 is becoming a major goal in T21 research. A comprehensive investigation of genes and miRNAs in the whole genome may improve our understanding of the genetic-epigenetic interactions of T21.

In this study, we investigated the genes and miRNAs with abnormal expression in placentas from T21 fetuses compared with that in euploid fetuses and found 17 genes that were negatively regulated by miRNAs in the T21 placentas. Among them, seven genes had increased and 10 had decreased expression. Of the seven genes with increased expression, four genes were on chromosome 21 and were target genes of three down-regulated miRNAs in the T21 placenta. The 10 genes with decreased expression in the T21 placenta were located on the various chromosomes and were target genes of five up-regulated miRNAs. Half of the eight miRNAs of interest are on the X-chromosome. Most of the tested samples were from males (Table 1). In male spermatogenesis, epigenetic changes play a crucial role in meiotic sex chromosome inactivation (MSCI) and escape. Escape from MSCI characterizes a set of miRNA genes such as mir-221, mir-374, mir-470 and mir-741 [29]. Up to 86% of the X-linked miRNAs escape MSCI during male spermatogenesis [30]. This is likely to have little impact in association between gene and miRNA expression of tested male samples.

Additionally, by in silico pathway-based exploratory analysis, we found an interaction network of three up-regulated genes (*U2AF1, DYRK1A*, and *KSR1*) and four down-regulated genes (*MRPL43, F2RL1, TICAM2,* and *MAP3K5*) in the T21 placenta. Our network showed the possibility of the processes involving *DYRK1A* and *MAP3K5*, being in the genetic-epigenetic mechanisms related to T21 pathophysiology. The DYRK1A protein is a member of the dual-specificity tyrosine-regulated kinases (DYRKs), and has the ability to phosphorylate serine/threonine and tyrosine residues. Its gene is located in the Down syndrome critical region of chromosome 21. DYRK1A overexpression alters both the phosphorylation of tau and alternative splicing factor, and causes an imbalance of 3R- and 4R-tau in the T21 brain [31]. In neurons, the hyperphosphorylation and accumulation of tau into neurofibrillary tangles (NFTs) was found to characterize some neurodegenerative disorders, known as taupathies, Alzheimer’s disease being among them [32]. Tau has an important impact on the organization of the cytoskeleton in neurons and, in particular, in the regulation of axonal transport. Therefore, it is considered a strong candidate gene for the neuronal degeneration associated with Down syndrome [33]. Interestingly, researchers have identified molecules that can modulate splicing, selectively targeting DYRK1A and cyclin-dependent kinase-like 1 [34, 35], thus opening up new avenues for T21 therapy. Mitogen-activated protein kinase kinase kinase 5 (MAP3K5) acts as an essential molecule of the MAPK signal transduction. It plays an important role in the cascades of cellular responses evoked by changes in the environment, and mediates the signals that determine cell fate, such as differentiation and survival. In particular, by activating MAPKs, MAP3K5, mediates signaling pathways involved in both the differentiation and survival of neuronal cells. MAP3K5-null mice show impairment of long-term recognition memory, in addition to hyperactivity in a novel environment, and superior motor coordination [36]. Therefore, MAP3K5 seems a good candidate for explaining the mechanisms underpinning intellectual disability and epilepsy [37]. Our results showed that miRNAs negatively regulated the expression of their target genes *DYRK1A* and *MAP3K5* in T21, likely through their transcriptional regulatory mechanisms of translational repression or mRNA degradation. Thus, our finding suggests that expression changes of miRNAs that target DYRK1A and MAP3K5 could lead to the changed levels of these two genes in T21, thereby playing a key part in the role of *DYRK1A* and *MAP3K5* in T21 pathogenesis.

In this study, different CVS samples were used for gene expression profiling and for miRNA expression analysis. The amount of material from CVS available for analysis was limited. Because fetal placenta samples at the first-trimester pregnancy were very difficult to obtain, a small amount of chorionic villus were obtained per case. Therefore, as this study was limited by its small sample size, a larger-scale study is needed to clarify the findings.

## Conclusions

To our best knowledge, this is the first study to survey whole genes and miRNAs in placentas of T21 fetuses. This study shows that 17 genes and eight miRNAs were differentially expressed between euploid and T21 fetuses and they were negatively regulated in T21. Furthermore, our results propose that many biological pathways that have been implicated in T21 and its complications are possibly regulated by these genes. Therefore, the present work provides a variety of information that may give to a better understanding of genetic-epigenetic modulations in T21.

## Abbreviations

CVS: Chorionic villus sampling
miRNA: microRNA
T21: Trisomy 21
